# Association between complete corpus callosum agenesis and recent-onset schizophrenia: A case report

**DOI:** 10.1192/j.eurpsy.2021.655

**Published:** 2021-08-13

**Authors:** B. Abassi, F. Fekih-Romdhane, H. Maktouf, M. Cheour

**Affiliations:** 1 Psychiatry E, Razi hospital, Manouba, Tunisia; 2 Psychiatrie Ibnomran, Razi Hospital, mannouba, Tunisia

**Keywords:** recent-onset schizophrenia, Association, corpus callosum agenesis

## Abstract

**Introduction:**

Corpus callosum agenesis (CCA) is a rare congenital disorder in which there is a partial or complete absence of the corpus callosum. Within the framework of an anatomoclinical approach to schizophrenia, a few studies have focused on its association with neurological disorders, including CCA.

**Objectives:**

In this perspective, we report a rare case of an adolescent with intellectual disability, with whose complete CCA was revealed by a recent-onset schizophrenia.

**Methods:**

A case report and literature review.

**Results:**

The teenager M.A. is from a first degree consanguineous marriage. He had no family history, no special habits and no history of seizures. Since early childhood, he had had a psychomotor acquisition delay and relationship difficulties that tended to worsen later. At school, he was teased by his peers and failed 4 times in different rows. At the age of 15, he dropped out of school. Verbal and physical aggressiveness, soliloquy, unmotivated laughter and vague ideas of persecution suddenly appeared, hence his psychiatric consultation one month later. Neuropsychological tests indicated current low Intelligence Quotient (60). Brain imaging revealed complete ACC (see Figure 1). He was diagnosed with schizophrenia and put on antipsychotic treatment at adequate doses and durations, with poor therapeutic response.

**Conclusions:**

Our observation provides additional support for neurodevelopmental models of schizophrenia, and confirms literature data indicating that severe structural brain abnormalities would lead to early onset psychotic symptoms which are often refractory to pharmacological treatments

